# Sonoporation by microbubbles as gene therapy approach against liver cancer

**DOI:** 10.18632/oncotarget.25875

**Published:** 2018-08-14

**Authors:** Luca Rinaldi, Veronica Folliero, Luciana Palomba, Carla Zannella, Rachele Isticato, Raffaele Di Francia, Massimiliano Berretta, Ilario de Sio, Luigi E. Adinolfi, Giancarlo Morelli, Secondo Lastoria, Lucia Altucci, Carlo Pedone, Massimiliano Galdiero, Gianluigi Franci

**Affiliations:** ^1^ Department of Medical, Surgical, Neurological, Metabolic and Aging Science, University of Campania “Luigi Vanvitelli”, Naples, Italy; ^2^ Department of Experimental Medicine, University of Campania “Luigi Vanvitelli”, Naples, Italy; ^3^ Department of Biology, Federico II University, Naples, Italy; ^4^ Department of Hematology, National Cancer Institute, Foundation G. Pascale IRCCS, Naples, Italy; ^5^ Department of Medical Oncology, National Cancer Institute IRCCS, Aviano, Italy; ^6^ Department of Precision Medicine, University of Campania “Luigi Vanvitelli”, Naples, Italy; ^7^ Department of Pharmacology, Federico II University, Naples, Italy; ^8^ Department of Diagnostic Imaging, Radiation and Metabolic Therapy, National Cancer Institute, Foundation G. Pascale IRCCS, Naples, Italy

**Keywords:** microbubbles, sonoporation, ultrasound, liver cancer, gene therapy

## Abstract

**Introduction:**

An innovative method, known as sonoporation, was used to induce the expression of silenced genes, such as (but not restricted to) TRAIL and p53, in liver cancer cells (HepG2). The principal aim of the present study was the re-activation of silenced apoptotic pathways in liver cancer models, by using diagnostic synovial microbubble as plasmid gene delivery tools in combination with epigenetic treatments.

**Material and methods:**

HepG2 cells were used as a liver cancer model. Microbubbles (Sonovue^®^) were chosen as gene deliver system in combination with the sonoporation approach. Plasmid pEGFP-TRAIL and pEGFP-p53 were selected and propagated in *Escherichia coli* grown in LB broth, in order to obtain the necessary amount.

**Results:**

Sonoporation was induced by using transducer (Sonitron 2000) and, among the several conditions tested, 3 MHz, 51% Duty Cycle, and 5 W/cm2, 30 s resulted as the best parameters. Data collected showed a dose dependent effect in terms of output energy. A transfection efficacy of 30 – 50% was achieved and recombinant gene expression induced apoptotic effects. In order to increase efficacy, we used the histone deacetylase inhibitor (HDACi, entinostat) MS-275, able to activate TRAIL and thus inducing a stronger pro-apoptotic effect in combination with TRAIL-gene re-expression.

**Conclusion:**

For the first time, it was shown the possibility to induce the exogenous expression of the pro-apoptotic gene TRAIL and p53 in a liver cancer HepG2 cells via a sonoporation procedure. The epigenetic treatment using HDACi was able to increase the pro-apoptotic effects of the gene therapy.

## INTRODUCTION

Ultrasound contrast agents are routinely used for diagnostic purposes in several fields of medicine, especially oncology [1]. One of the most common commercial formulation to achieve this goal is mainly represented by SonoVue^©^ (Bracco, Milan, Italy) [2], a solution of sulfur hexafluoride microbubbles (diameter 1-6 μm) encapsulated by a phospholipid membrane, which prevents its fast dissolution in blood. Recently, therapeutic applications of microbubbles have been rising, such as: i) drug delivery [3]; ii) gene delivery [4]; iii) antibodies targeting [5]. This capability is enhanced in case of a combination of microbubbles with sonoporation [6]. This process is obtained by subjecting microbubbles to acoustic pressures (MI >0.3-0.6) able to produce a mechanic stress driving to the membrane collapse [7]. Microbubbles destruction induces a microstream/local microjet around the bubbles, which results in an increase in permeability among adjacent cell membranes [8]. The mechanism of pore formation in cell membranes is believed to facilitate the entry into the cell of molecules such as plasmid DNA and oligonucleotides [9]. Sonoporation and its applications have been used and validated in several models, *in-vitro* cell models and *in-vivo* pre-clinical studies [6].

Liver cancer represents one of the leading causes of death in developed countries [10]. In the last years, epigenetic mechanisms have been assuming important roles in liver cancer etiology. In this scenario, for instance, a recent study showed how genome methylation level in liver tissue cancer compared to the healthy proximal tissue of the same patients is aberrant. There is a direct correlation between methylation level and the aberrant liver cancer expression of genes such as AJAP1, ADARB2, PTPRN2, SDK1 [11]. Methylation does not represent the only epigenetic mechanism involved in liver cancer initiation, progression and fate. Regulation and deregulation of; i) post translational modification on histone and not histone target; ii) micro-RNAs (miRNAs); iii) long noncoding RNAs (lncRNAs) are only part of epigenetic phenomena underlying the liver cancer pathology [11, 12]. Moreover, down regulation of pro-apoptotic factors, such as p53 and TRAIL, are considered genetic deregulation events leading normal hepatocytes to transform into cancer cells [13]. P53 plays a fundamental role in liver cancer disease initiation, progression and responsiveness [14]. TRAIL is directly involved in stress response and pro-apoptotic pathway [15]. While gene therapy is still far from a common therapeutic use, due to the safety issues, sonoporation may represent an alternative route allowing delivery of suicide genes like p53 and TRAIL in liver cancer cell. Currently, a small molecule, Sorafenib has been approved for hepatocellular carcinoma (HCC) first line treatment [16]. It inhibits serine-threonine kinases, C-Raf and B-Raf and the angiogenesis by inhibiting the receptor tyrosine kinase activity of the vascular endothelial growths and factor receptors (VEGFR) 1–3, platelet derived growth factor receptor (PDGFR)-beta. A recent phase III clinical trial showed the non-inferiority of lenvatinib with sorafenib as first-line chemotherapy for HCC [17]. It also inhibits angiogenesis via VEGFR 1–3, PDGFR-alpha, and fibroblast growth factors receptor (FGFR). Regorafenib is an oral multikinase inhibitor (VEGFR 1–3, PDGFR-β, FGFR, RAF-1, B-Raf) implicated in angiogenic and cancer growth-promoting pathway and approved for the second-line treatment in patients who failed on sorafenib [16].

Recently, a tyrosine kinase inhibitor, cabozantinib, showed promising results in a phase III trial and is proposed as the second-line treatment option in patients with advanced HCC [18]. In addition, promising results showed Nivolumab, which interferes with the PD-1 receptor restores T-cell–mediated anti-tumour activity [16].

Despite these challenging therapeutic alternatives, novel treatment regimens are in urgently needed.

In this scenario, microbubbles-based delivery strategy may represent an alternative approach for gene delivery to contrast liver cancer. In the present manuscript, we showed the reactivation of silenced apoptotic pathways in liver cancer cells, by using diagnostic synovial microbubbles as a plasmid gene delivery tool.

## RESULTS

**Figure 1:**
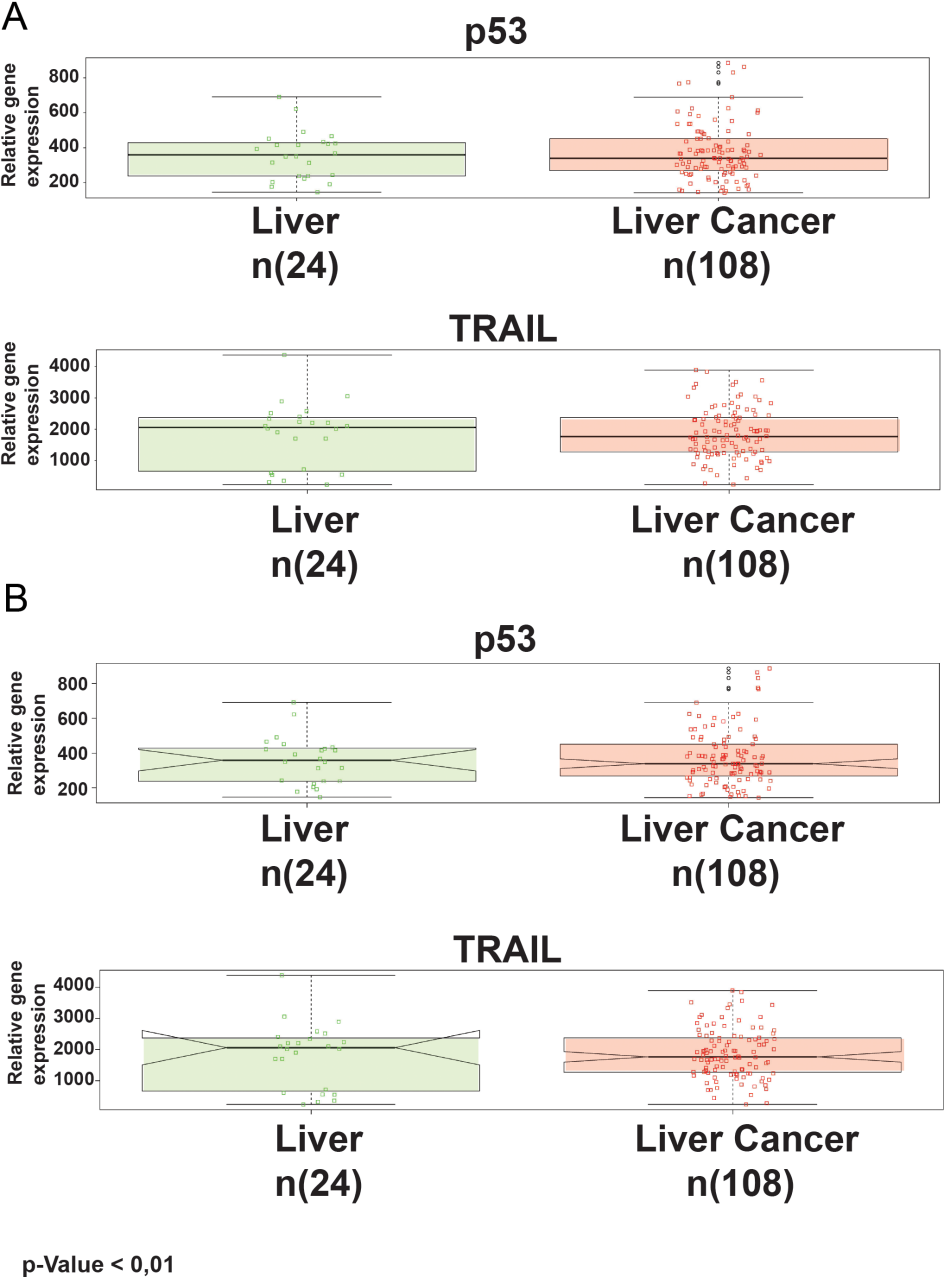
Standard box-whisker plots of p53 and TRAIL expression in healthy and cancer tissues **(A)** The green boxes are representative of healthy tissues, meanwhile the red boxes are for the cancers. The notches on the sides of the boxes **(B)**.

Key gene regulators are often correlated with tumor initiation, progression and fate. In this scenario, we investigated the relative abundance of two master genes in tumor regulation: p53 and TRAIL. Medisapiens database (http://ist.medisapiens.com/) was used for their evaluation in liver cancer patients compared to healthy. Results are shown in Figure 1.

**Figure 2:**
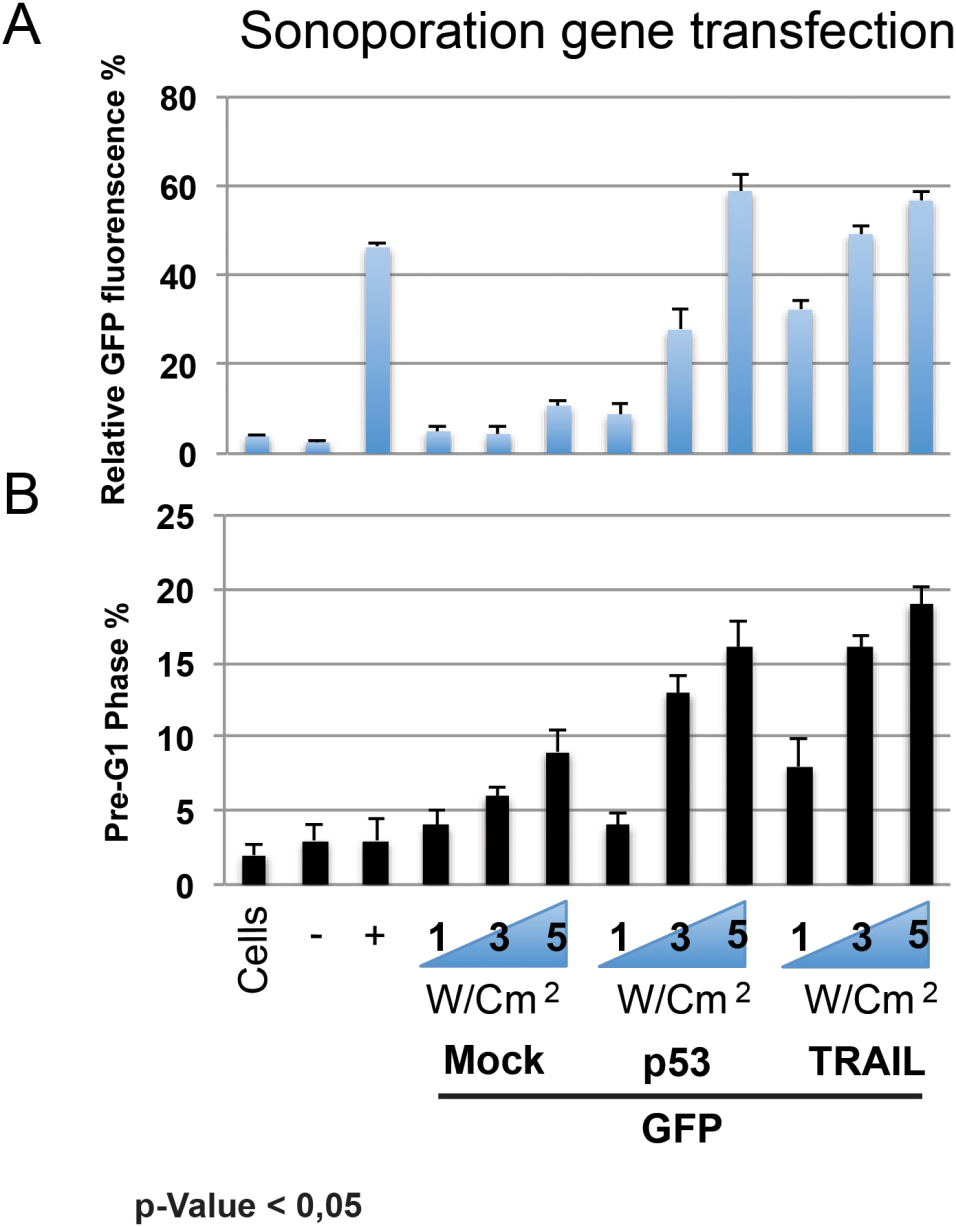
Protein (GFP) construct with several combinations in term o MHz, Duty Cycle and W/cm2 were tested Efficacy of sonoporation was reported as relative GFP-fluorescence in histogram graph **(A)**. FACS analysis of Pre-G1 phase **(B)**.

The analysis output highlights, through box-whisker plots, a higher expression of p53 and TRAIL expression in healthy (green) than in cancer tissues (red). In Figure 1B, the notches on the sides of the boxes (another statistical analysis graphical visualization) underline how they do not overlap. If two notches do not overlap, this means that there is a significant difference between the medians of the two groups (the 25th percentile is located at the bottom of the box, while the 75th percentile is at the top; the horizontal line represents the median). The whiskers extend to 1.5 times the interquartile range from the edges of the box, and any data point beyond this is considered an outlier, marked by hollow circles. The Phenoplot shows a heat map of p53 and TRAIL expression along liver cancer patients, and associated clinical data, within the selected dataset. In order to achieve the best experimental condition, GFP constructs were tested with several combinations in term of power, measured by MHz, Duty Cycle and W/cm2. Among all several conditions, some of them showed a dramatically toxic effect. In detail, we observed a higher toxic effect at 5 MHz, 100% Duty Cycle, and 3 W/cm2, 60s with over than 80% cells in Pre-G1 phase. Meanwhile, parameters such as 1 MHz, 30% Duty Cycle, and 1 W/cm2 60s were not sufficient for allowing an efficient gene delivery. Sonoporation efficacy was reported as relative GFP-fluorescence in the histogram graph (Figure 2A). At the same time, we monitored the pre-G1 phase via FACS analysis (Figure 2B). Results highlight a strong correlation between sonoporation efficacy and the increase of toxicity. Based on this postulate, we found that the 3 MHz, 50% Duty Cycle, and 5 W/cm2 for 60s were able to attain a good gene transfection efficiency and limited toxicity (less than 20%). The results are representative of three independent experiments, and stringent statistical filters were applied (p-value < 0,05).

In our view, the gene therapy via microbubbles sonoporation should be integrated with the use of epigenetic modulators.

**Figure 3:**
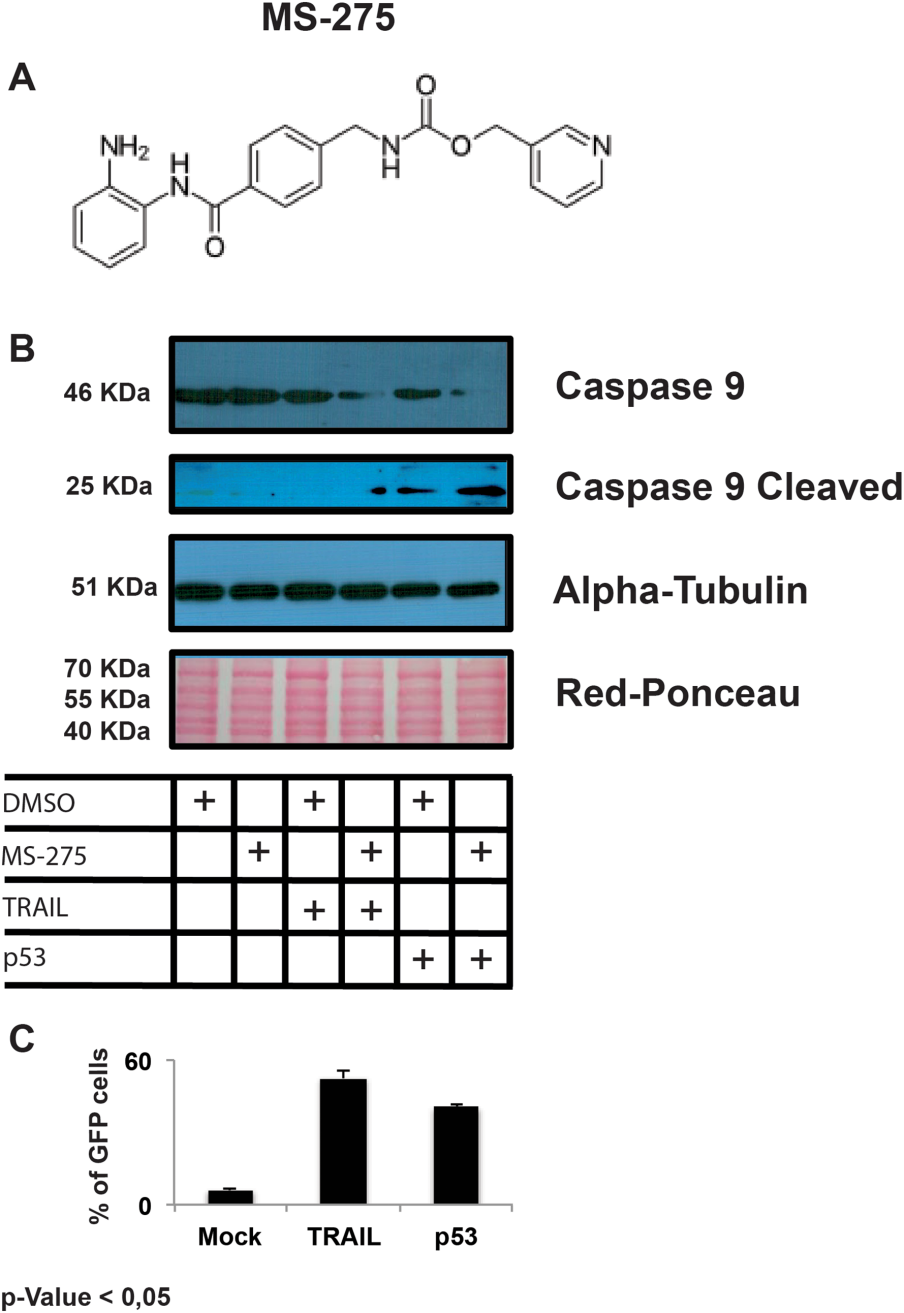
Structure of Entinostat **(A)**. Western-blot analysis of Caspase 9 cleavage activation mediated in HepG2 cell line treated with Entinostat (MS-275) **(B)**. The respective % of cells expressing the recombinant proteins are reported ad histograms in the panel **(C)**.

In this scenario, we evaluated the apoptotic pathway activation mediated by gene re-expression in HepG2 cell line treated with Entinostat (MS-275) (Figure 3A). Cells were treated for 6h with Entinostat post 24h from sonoporation. This experimental scheme takes into consideration the fact that after 6h the epigenetic modulator Entinostat starts to act on chromatin remodelling and gene expression. Caspase 9 cleavage activation was detected by western blot and reported in Figure 3B. The respective percentages of cells expressing the recombinant proteins are reported as histograms in the Figure 3C. All data of Figure 3 panel are the representation as means of three independent experiments with statistical significance P-value < 0.05.

## DISCUSSION

Among human cancers, HCC registers a high incidence rate with patients comorbidity and a high cost for the society, in economic and social terms [19]. HCC may be related to either a viral [20] or non-viral [21, 22] aetiology, and prevention may not be sufficient to reduce the burden.

In this view, the search of new cancer treatments and the optimization of patients stratification is mandatory. Gene expression evaluation currently represents a master route in patient’s treatment. Experimental evidence highlights how treatment’s failure is not commonly due to the inefficacy of the used drugs, while it is highly dependent on the genetic and epigenetic factors. On the other hand, recent efforts striving for innovative treatment of cancer patients highlights the possibility to act on epigenetic mechanisms in order to achieve a biological response against either deregulated or silenced cancer cells. Epigenetic phenomena are, together with gene integrity, mandatory for the homeostasis of cells and our organism. Nowadays, it is becoming evident that several human diseases are strongly based on gene/chromosome alterations and epigenetic deregulations. For instance, only a few years ago the first epigenetic treatment for a peculiar lymphoma with the Merck drug Vorinostat was approved. Actually, hundreds of clinical trials with epigenetic modulators are on-going. Despite the effort of scientists and physicians, the cure of cancer is still far away and the future of cancer treatment seems to be personalized medicine. In this horizon, we address the possibility to use an innovative method for non-viral gene delivery in cancer cells in combination with epigenetic treatment.

Results showed for the first time the possibility to induce the exogenous expression of pro-apoptotic gene TRAIL and p53 in a liver cancer model HepG2 via sonoporation procedure with the epigenetic treatment modulator MS-275. We recorded an enhanced and synergic effect of pro-apoptotic events with the combination of gene sonoporation and epigenetic treatment. The efficacy of Entinostat was already confirmed in previous studies, either as anticancer drug [23–26] inducer of differentiation [27] senescence [28] or anti-angiogenesis effector [29, 30].

Despite the significant advancement in this area in the most recent years, several technical measures would be necessary to increase the amount of gene transfection and its enhancement.

In fact, in our experience, the increase in transducer power and timing of exposition, leading to a major transfection, was limited by the cell damage. Moreover, the heterogeneous “Sonovue microbubbles” are not specific for laboratory tests dealing with cellular material; however, although we used unsuitable microbubbles, our tests were useful to evaluate the efficacy of the combination of epigenetic and transfection factors in pro apoptotic effects.

Recently, Manta *et al.* [31] suggested the use of cationic microbubbles complexed with a small length mini-plasmid DNA to improve gene transfection of hepatocytes and long term liver expression. This study confirmed how the optimization of ultrasound parameters and of the chemical and electrostatic structure of the microbubbles could play a pivotal role in increasing the efficacy of gene delivery. Moreover, the sonoporation method by Sonovue has already provided encouraging results in a recent human trial, whereas the gemcitabine treatment in inoperable pancreas cancer has been improved, extending the survival of patients without additional toxicity [32].

In conclusion, gene delivery by microbubbles/ultrasound represents an emerging field to improve cancer therapy. Our experience showed the efficacy of the delivery in producing pro apoptotic effects on the cancer liver cells in a view of the epigenetic treatment.

The future goal will be the translation of our system into animal models, in order to evaluate the *in-vivo* effect of a new protocol for epigenetic and plasmidic gene expression in cancer therapy.

## MATERIALS AND METHODS

### Cell culture

The HepG2 ATCC (HB-8065) cell line was used in cell-based experiments. The cells were disseminated and expanded as reported in the manufacture guideline (ATCC). The cells were propagated with Eagle's Minimum Essential Medium, (Catalog No. M4655-500ML MERCK) implemented with 10% fetal bovine serum (FBS) (Euroclone), 2 mM L-glutamine (Euroclone) and antibiotics (100 U/ml penicillin, 100 lg/ml streptomycin) (Euroclone).

### Statistical analysis

All biological experiments were performed in three individual replicates and the results were consider significant with a p-value < 0.05. Bioinformatics analysis take in consideration that all the original dataset was filtered and statistically approved by mediasapiens platform and differences with p-value < 0.01 were considered significant.

### Transfection

Microbubbles (Sonovue^®^) were used at standard condition, according to manufacturer’s instructions. pEGFP-TRAIL plasmid (#10953 Addgene) and pEGFP-p53 (#12091 Addgene) were transformed and amplified in Escherichia coli DH5α competent cells (Invitrogen) using the relative antibiotics for the transformants selection. Selected clones were incubated overnight at 37°C and 200 rpm in LB Broth (Miller). Plasmids were purified using Thermo-Fisher PureLink (Thermo-Fisher scientific Cod. K210017). Plasmids concentration and quality were verified via Nano Drop (ND-1000) (Thermo Fisher Scientific) at 260 nm and with the restriction enzymatic digestion as suggested in the respective genetic map.

The microbubble were mixed with plasmid (1μg/5x10^6^microbubble) in OptiMEM (Gibco, Life technologies Cod. 31985-062) and incubated at room temperature with gentle shaking for 20 minutes. Transfections were achieved by an ultrasound device (Sonitron 2000, Artison corporation^®^) and compared with standard protocols using lipofectamine 2000 (Cod. 11668027 Thermofisher) as fully described in the result section.

### FACS acquisition and analysis

HepG2 cells were transfected with TRAIL-GFP and p53-GFP. The Green Fluorescent Protein expression was analysed via FACS Excalibur (BD).

In details, the cells washed twice time with PBS (Euroclone) 48h after sonoporation and harvested with trypsin solution (GIBCO) the cell, than the trypsin was removed via centrifugation at 1200 rpm and the cell pellet was resuspended in PBS1X. The cell cycle analysis was achieved via FACS analysis and results were analysed with.Cell-Quest and ModFit software (Verity). In briefly, the cells were washed in Phosphate Buffer Solution (PBS1X) and and re-suspended in Cell Cycle Buffer (Propidium Iodide Staining Solution: 3.8 mM sodium citrate, 50 ug/ml PI (Sigma, P 4170). Finally, the RNase A stock solution was used: 10 ug/ml RNase A (Worthington Biochemicals, RASE LS005649, LS005650).

### Protein extract

Cell protein extract and western blot analyses were conducted as described in Franci *et al.* [33]. Briefly, the cells were washed in PBSX1, the cells pelleted were resuspended in lysis buffer (50 mM Tris-HCl pH 7.4, 150 mM NaCl, 1% NP40,10 mM NaF, 1 mM PMSF and protease inhibitor cock-tail). The cell extract was obtained with an incubation of 20 min at 4°C. Cellular debrits were removed via centrifugation at max speed for 30 min. at 4°C. The protein extract was measured by Bradford assay (Bio-Rad, Hercules, CA, USA). The 10% acrylamide (BIORAD) gel was loaded with 50 μg of total protein extract. The proteins were transferred on nitrocellulose filters and than, stained with Red Ponceau (Sigma Aldrich).

The nitrocellulose filters were incubated with the primary antibodies alpha-tubulin at the final concentration of 0,5 μg/ml as reported in the official data sheet (Sigma T6074) and caspase 9 diluted 1:1000 in TBS-T1X as reported in the official data sheet (Cell Signalling #9502) and incubated for 2h at R.T in agitation. The secondary antibodies were acquired from GE and diluted 1:1000 in TBS-T1X 3% milk powder (BIORAD) and incubated for 45 min at R.T. in agitation.

X-ray Film for Western Blot Detection (Cod. 34090 ThermoFischer) were used for signal acquisition post ECL (Cod. 32106 ThermoFischer) incubation.

For the epigenetic treatment we used the inhibitor for Class I HDACs, MS-275, also known as Entinostat (Selleckchem cod. S1053). Cells were treated for 6h with Entinostat post 24 from sonoporation.
