# Correction: Sonoporation by microbubbles as gene therapy approach against liver cancer

**DOI:** 10.18632/oncotarget.26305

**Published:** 2018-10-30

**Authors:** Luca Rinaldi, Veronica Folliero, Luciana Palomba, Carla Zannella, Rachele Isticato, Raffaele Di Francia, Massimiliano Berretta, Ilario de Sio, Luigi E. Adinolfi, Giancarlo Morelli, Secondo Lastoria, Lucia Altucci, Carlo Pedone, Massimiliano Galdiero, Gianluigi Franci

**Affiliations:** ^1^ Department of Medical, Surgical, Neurological, Metabolic and Aging Science, University of Campania “Luigi Vanvitelli”, Naples, Italy; ^2^ Department of Experimental Medicine, University of Campania “Luigi Vanvitelli”, Naples, Italy; ^3^ Department of Biology, Federico II University, Naples, Italy; ^4^ Department of Hematology, National Cancer Institute, Foundation G. Pascale IRCCS, Naples, Italy; ^5^ Department of Medical Oncology, National Cancer Institute IRCCS, Aviano, Italy; ^6^ Department of Precision Medicine, University of Campania “Luigi Vanvitelli”, Naples, Italy; ^7^ Department of Pharmacology, Federico II University, Naples, Italy; ^8^ Department of Diagnostic Imaging, Radiation and Metabolic Therapy, National Cancer Institute, Foundation G. Pascale IRCCS, Naples, Italy

**This article has been corrected:** The correct Acknowledgement information is given below:

**ACKNOWLEDGMENTS**

Blueprint 282510; MIUR20152TE5PK; COST-H2020: EPICHEMBIO CM1406; AIRC-17217; RISE-H2020: Ocean Medicine, PROGRAMMA VALERE provided the publication fee for the manuscript

Original article: Oncotarget. 2018; 9:32182-32190. https://doi.org/10.18632/oncotarget.25875

